# Supplementary material for: The Effect of Internal and external imagery on learning badminton long serve skill: The role of visual and audiovisual imagery
Source: PLoS One. 2024 Sep 19;19(9):e0309473. doi: 10.1371/journal.pone.0309473 (PMC11412531; doi:10.1371/journal.pone.0309473)
Supplement: Attachment — Submitted filename: Review of PLOS ONE-D-23-43363.docx [file pone.0309473.s002.docx]

**Referring to PONE-D-23-43363**

**Title:** The Effect of Internal and External Audiovisual Imagery on Learning Badminton Long Serve Skill

This study aims to investigate the effect of internal and external audiovisual imagery on learning badminton long serve skill. It is explores the influence of different motor imagery methods on motor learning and performance. It's a very interesting topic and valuable for educational implications in sport. The unique auditory model used in this research (based on which the characteristics kinematics of the movement has been converted into sound) and its use in motor imagery is the new approach of this research in motor imagery. Below are some suggestions that should be considered by the authors of the manuscript:

**Abstract**

In the abstract of the manuscript, the result of comparing the internal and external imagery groups should be reported.

**Introduction**

In the introduction, it is suggested to refer to the researches that have investigated the effect of sonification-based intervention on performance and motor learning.

**Research Method**

- In line 202, refer to the source of sonification sandbox software
- It is better to present the research protocol in the form of a table

**Research findings**

In the findings section, a number of effect sizes not reported. Be sure to report effect sizes for all pairwise comparison.

**Discussion and conclusion**

The discussion of this manuscript is well written and the explanation of the results is good based on previous researches and the proposed hypotheses. It seems that the effect of the intervention in this research is first on the quality of motor imagery and then on performance and motor learning. Therefore, it is better to mention this topic at the end of the discussion.
